# Iron gets in the way

**DOI:** 10.7554/eLife.90743

**Published:** 2023-08-10

**Authors:** Kuldeep Singh, Rajeev Malhotra

**Affiliations:** 1 Division of Cardiology, Department of Medicine, Massachusetts General Hospital, Harvard Medical School Boston United States

**Keywords:** atherosclerosis, iron metabolism, postmenopause, estrogen receptor α, hormone replacement therapy, Mouse

## Abstract

Accumulation of iron with age may inhibit the benefits of hormone replacement therapy on cardiovascular disease in late postmenopause.

**Related research article** Xu T, Cai J, Wang L, Xu L, Zhao H, Wang F, Meyron-Holtz EG, Missirlis F, Qiao T, Li K. 2023. Hormone replacement therapy for postmenopausal atherosclerosis is offset by late age iron deposition. *eLife* **12**:e80494. doi: 10.7554/eLife.80494.

Menopause is a biological process often occurring between ages 45 and 55, when egg production ceases and the menstrual cycle ends due to a decline in reproductive hormones, especially estrogen. The postmenopausal state increases the risk of cardiovascular diseases and dying from cardiac-related illnesses, such as a heart attack or stroke. For instance, previous work has shown that compared to age-matched men, premenopausal women are less prone to atherosclerosis, a formation of plaques made up of fatty deposits and other substances inside arteries. However, this protection is lost after menopause, with atherosclerosis progression being particularly prominent in late postmenopausal women over the age of 65 (Moss et al., 2019).

Some studies have suggested that the primary method for treating the symptoms of menopause, hormone replacement therapy (HRT), can also improve cardiovascular health (Herrington et al., 1999; Stampfer and Colditz, 1991). While others have reported HRT to have the opposite effect and actually increase the risk of cardiovascular disease (Hulley et al., 1998; Rossouw et al., 2002). One potential reason for these contradicting results is that HRT benefits cardiovascular health in the early stages of postmenopause but not later on (Boardman et al., 2015; Hlatky et al., 2002; Hodis et al., 2016; Rocca et al., 2014). Now, in eLife, Tong Qiao, Kuanyu Li (both at Nanjing Medical University) and colleagues – including Tianze Xu and Jing Cai as joint first authors – report what could be causing this age-related effect (Xu et al., 2023).

The team (who are based in China, Israel and Mexico) collected plaques and blood samples from atherosclerosis patients who were also postmenopause and divided them into two groups: early postmenopausal (55–65 years old) and late postmenopausal (over 65). They found that samples from the late group contained higher levels of iron and ferritin, the protein that stores iron inside cells. Late postmenopausal patients also produced less of the receptor ERα, which binds to estrogen and is critical for maintaining healthy blood vessels. This suggests that age-related accumulation of iron is negatively associated with ERα levels.

Next, Xu et al. carried out experiments in young mice that had been genetically modified to have atherosclerosis and also had their ovaries removed to trigger the symptoms of menopause. Treating the mice with a form of estrogen known as estradiol, which is used in HRT, increased ERα levels, but only in mice that were early postmenopausal. Conversely, late postmenopausal mice treated with estradiol had less ERα compared to non-treated groups. Thus, it appears that the high levels of iron in the tissues of late postmenopausal mice is somehow impairing the effects of estradiol.

To confirm this effect, Xu et al. conducted further experiments on atherosclerosis mice lacking the gene for ferroportin 1, specifically in their myeloid cells (a type of cell that will mature into certain immune cells of the blood, such as macrophages). The removal of ferroportin 1, a transmembrane protein that exports iron, leads to a buildup of iron inside macrophages. Iron-overloaded macrophages have been shown to speed up the progression of atherosclerosis, whereas macrophages with low levels of iron reduce progression of the disease (Cai et al., 2020; Malhotra et al., 2019). When the mutant mice were treated with estradiol, this resulted in larger atherosclerotic lesions in both the early and late stages of postmenopause. This suggests that iron influences how estradiol impacts atherosclerosis. The mice also had lower levels of ERα in their aortas, which was reduced even further following estradiol treatment.

Xu et al. found that high amounts of iron combined with estradiol downregulated ERα by activating Mdm2, a protein that induces ubiquitin degradation of the estrogen receptor (Figure 1). Reducing the expression of the gene coding for Mdm2 through iron chelation therapy restored ERα levels and attenuated the development of atherosclerosis in late postmenopausal mice.

**Figure 1.**
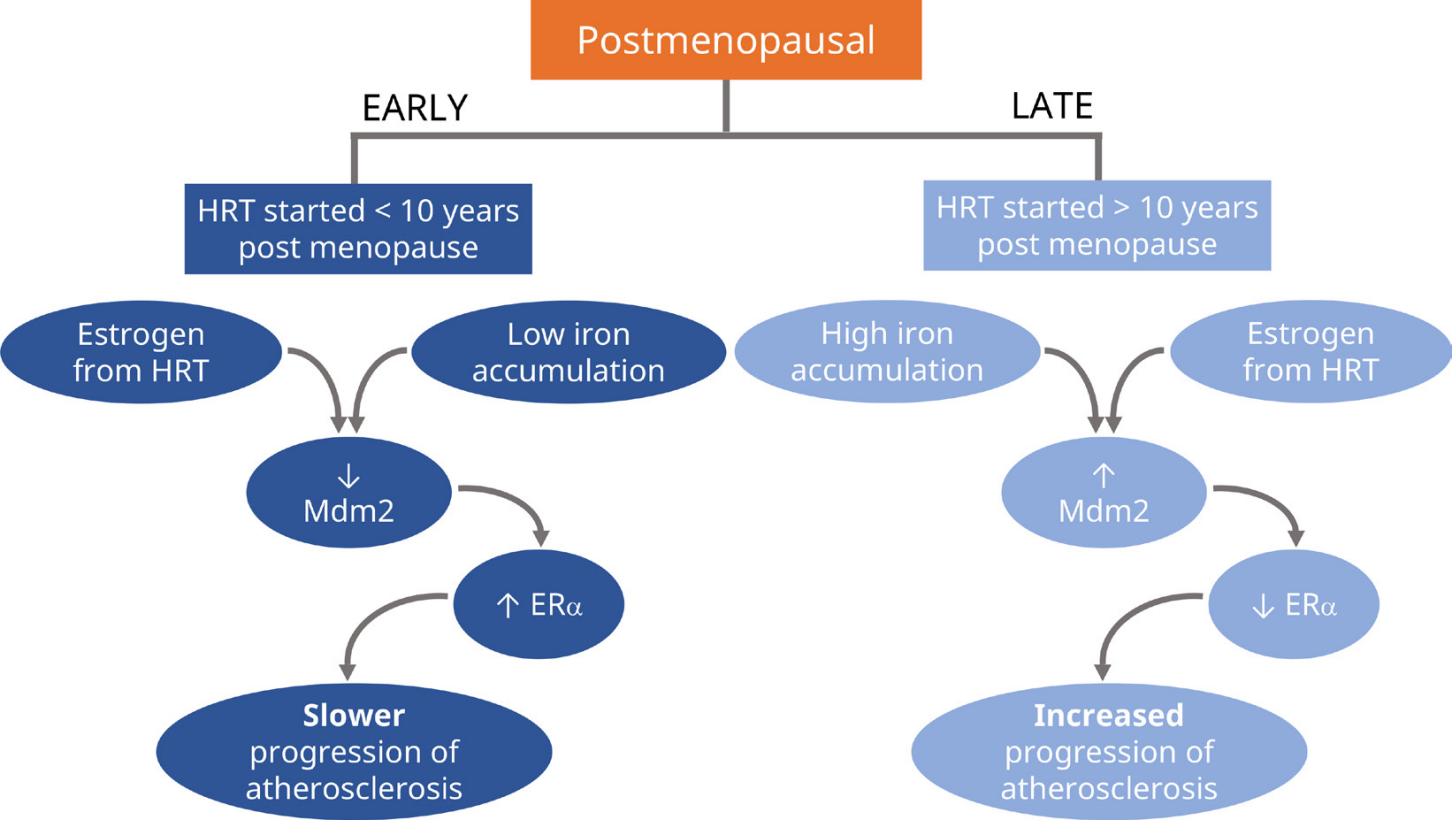
Schematic representation of the proposed divergent effects of HRT treatment in postmenopause. If hormone replacement therapy (HRT) is prescribed in the early stages of postmenopause (first 10 years), the estrogen from HRT will slow the progression of atherosclerosis (left; dark blue). Experiments in mice suggest it does this by reducing the levels of Mdm2, an enzyme that signals for the degradation of certain proteins. This results in higher levels of the receptor for estrogen, ERα, which also helps to maintain healthy blood vessels. HRT has the opposite effect when prescribed during the later stages of postmenopause (10 years or more), where it will increase the progression of atherosclerosis (right; light blue). Xu et al. propose that this is due to older individuals having higher levels of iron accumulating in their tissues, which causes estrogen to boost the amount of Mdm2 instead, resulting in lower levels of ERα and increased progression of atherosclerosis.

Overall, this study provides strong evidence that iron accumulation shifts estradiol from a drug that reduces atherosclerosis to a drug that promotes its progression. The excess iron that accumulates in older individuals promotes degradation of ERα, and this is then further amplified by estradiol treatment, counteracting the anti-atherosclerosis effects of the drug. It is important to note that this study does not directly address how HRT and iron affect the stability of atherosclerosis plaques or their chance of rupturing, which is often the cause of a heart attack or stroke.

Previous work has shown that HRT reduces mortality and coronary heart disease, but only if administered in the first 10 years post menopause (Boardman et al., 2015). The study by Xu et al. suggests that this effect may be due to iron accumulating in the blood with age. This highlights the need for clinical studies investigating how iron levels impact the benefits of HRT on cardiovascular health post menopause.
